# Near-ubiquitous presence of a vancomycin-resistant *Enterococcus faecium* ST117/CT71/*van*B –clone in the Rhine-Main metropolitan area of Germany

**DOI:** 10.1186/s13756-019-0573-8

**Published:** 2019-07-29

**Authors:** Linda Falgenhauer, Moritz Fritzenwanker, Can Imirzalioglu, Katrin Steul, Marlene Scherer, Rhine-Main VREfm study group, Sabine Albert-Braun, Klaus-Peter Hunfeld, Volkhard Kempf, Andreas Kneifel, Adnan Kukic, Bernhard Jahn-Mühl, Katharina Madlener, Klaus Oberdorfer, Jörn-Peter Oeltze, Jörg Schulze, Navid Sotoudeh, Rolf Tessmann, Ursel Heudorf, Trinad Chakraborty

**Affiliations:** 1 Institute of Medical Microbiology, Justus Liebig University Giessen and German Center for Infection Research (DZIF), Partner Site Giessen-Marburg-Langen, Schubertstrasse 81, 35392 Giessen, Germany; 2 Rhine-Main- Network on MDRO (multidrug-resistant organisms), Breite Gasse 28, 60313 Frankfurt/Main, Germany

**Keywords:** VRE, ST117, cgMLST, WGS

## Abstract

Whole-genome sequencing analysis of Vancomycin-resistant *Enterococcus faecium* isolates from the Frankfurt metropolitan region revealed that 78/94 isolates were MLST type ST117, cgMLST complex type CT71 with a common *van*B chromosomal insertion site. This indicates circulation of a single VRE clone in a catchment area of 5,000-km^2^ with 3 million inhabitants.

**Electronic supplementary material:**

The online version of this article (10.1186/s13756-019-0573-8) contains supplementary material, which is available to authorized users.

## Background

Vancomycin-resistant *Enterococcus faecium* (VREfm) are an important cause of nosocomial infections worldwide [1]. The WHO ranks VREfm on its high priority list of multidrug-resistant microorganisms because of increasing prevalence and transmission rates in community and healthcare settings [2]. Since 2014 there has been a dramatic increase of VREfm prevalence among clinical samples in Germany. Marked regional differences have been noted, with high VREfm prevalence within an east-west axis in central Germany (“VRE-belt”), that includes the German federal state of Hesse [3, 4].

The presence and impact of epidemic VREfm on individual patients entering the healthcare system particularly within the “VRE-belt” is poorly understood. Here we report on the genome-based analysis and comparison of VREfm isolated from patients with or without a prior history of hospitalization during admission to intensive care units or other wards with patients at risk for VREfm colonization/infection i.e. hemato-oncological and transplantation units.

### Sampling area, patient characteristics

Sampling was performed between November 2017 and June 2018 in 17 hospitals within the Frankfurt am Main metropolitan region, all of whom are members of the Network on multidrug-resistant organisms in the Rhine-Main area (MDRO Network Rhine-Main). The size of hospitals varied between 100 and 1488 beds. Among these, 11/17 were tertiary care hospitals, while the remaining six hospitals were either standard care (*n* = 3), general hospitals (*n* = 2) or a specialized clinic (*n* = 1) (Additional file 1: Table S1). Participating hospitals were requested to provide VREfm isolates from samples obtained from patients at admission (within 72 h) to intensive care units or other wards where patients with a high risk for VREfm colonization/infection were treated, i.e. hemato-oncological and transplant units (*n* = 85, anal/rectal swabs, stool specimens). An active admission screening of all patients or of defined risk patients was not performed in this study. Hence, determining the prevalence of VRE carriage at admission was not the purpose of this study. The number of isolates per hospital (Additional file 1: Table S1) was dependent on the size of the catchment area of the respective hospital. For hospitals that did not have the requested amount of VREfm-positive screening samples within the study period, VREfm from clinical samples were included (*n* = 10). These comprised of isolates from blood cultures, urine, wound smears, intra-abdominal surgery smears and a central venous catheter isolate. Identification and antibiotic resistance determination of VREfm was performed using standard laboratory methods and technologies (e.g. chromID VRE plates, MALDI MS, VITEK II, BioMérieux, Nürtingen, Germany) in the labs providing regular microbiological service for the participating hospitals.

In total, VREfm isolates from 95 patients were included. Patient meta-data was collected using a questionnaire (Table 1).

**Table 1:** Depiction of the patient meta-data

Parameter		n	%
Sex	Male	54	56.8
	Female	41	43.2
Age	<60	15	15.8
	60- < 70	21	22.1
	70- < 80	29	30.5
	> 80	29	30.5
	Not reported	1	1.1
Underlying disease	Hemato/oncology	19	20.0
	Cardiology	19	20.0
	Other	36	37.9
	Not reported	21	22.1
Travel abroad during the last 12 months	None	45	47.4
	Yes	8	8.4
	Indeterminate	42	44.2
Previous hospital stays within the last 12 month	No	13	13.6
	Yes	76	80.0
	Not ascertainable	6	6.3
Previous antimicrobial therapy*	Vancomycin	9	9.5
	Teicoplanin	6	6.3
	Piperacillin/Tazobactam	25	26.3
	Carbapenem	19	20.0
	Cephalosporin	20	21.1
	Penicillin	12	12.6
	Metronidazole	12	12.6
	Quinolone	17	17.9
	Treated with antibiotics	72	75.8
	No antibiotic treatment	2	2.1
	Not reported	21	22.1

*multiple answers were possible

The mean age of the patients was 71.2 ± 14.6 years, and ranged from a new-born to 95 years old. Fifty-four patients were male and 41 female. Information regarding a previous hospital stay during the last 12 months was reported in 93.6% (89/95) of the patients. Of these, 85% (76/89) reported a hospital stay. An underlying disease was reported in 77.9%. For 72/95 patients, an antimicrobial therapy during the past 12 months was recorded. Prior treatment with vancomycin was reported in 9.5%. VREfm was a first-time detection in 81 patients. Pre-existing VREfm carriage was known in 14 cases prior to the study.

### Characteristics of the VREfm isolates

VREfm were isolated and tested for susceptibility by the participating MDRO Network Rhine-Main centres using standard procedures for clinical diagnostic laboratories. VREfm isolates were generally were ampicillin-, fluoroquinolone- and carbapenem-resistant and linezolid-susceptible.

Whole genome sequencing (WGS) was performed as reported earlier [5, 6]. Resistance gene prediction, and Multilocus sequence typing (MLST) was performed using goseqit tools (https://www.goseqit.com/, Additional file 2: Table S2). Ninety-three VREfm harbored *van*B, and a single isolate harbored *van*A. One isolate did not harbor any *van* gene and was excluded from further analysis.

Analysis of the virulence genes was performed using goseqit tools. The presence of the enterococcal surface protein Esp required for promoting biofilm formation (Additional file 2: Table S2), and the PTS_clin_ phosphotransferase system associated with colonization potential of clinical isolates [7] as well as the uptake and utilization of amino sugars such as β-N-acetylglucosamine commonly found in mucin on the surfaces of epithelial cells and in biofilms were detected using blastn [8]. All *van*-encoding isolates harbored the *efaAfm* gene, suggested to be involved in cell wall adherence, which is concordant with the results from earlier studies [9]. Ninety-two isolates harbored the *hylEfm*, *acm* and PTS_clin_, while 90/94 isolates carried the *esp* gene.

Almost all isolates (90/94), regardless of source, were ST117 with the remaining four isolates each representing ST80, ST192, ST262 and ST1428. The ST262 isolate harbored a *van*A gene.

**Fig. 1:**
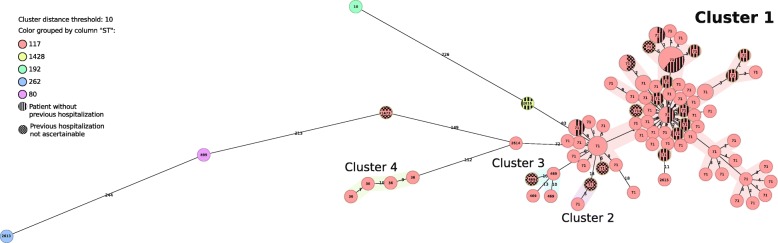
Minimum spanning tree of the sequenced VREfm isolates based on the cgMLST data. The figure was created according to reference [10]. Cluster distance threshold was set to 10 cgMLST alleles. Size of circles is according to number of isolates. Numbers in the circles indicate the complex type of the respective isolates. Colors of circles indicate multilocus sequence types

The use of MLST-based data to classify VREfm is controversial because of its high recombination rates that masks relatedness of otherwise highly related strains. Therefore, further differentiation of the ST117 isolates using a core-genome MLST (cgMLST) was performed (Ridom SeqSphere+ 5.1.0., Ridom GmbH, Münster, Germany; *Enterococcus faecium* scheme [10]). This analysis revealed that 78/90 (87%) of the ST117 isolates, i.e. from both non-clinical as well as clinical samples, were all members of a single cgMLST complex type (CT71, Fig. 1). Minor CTs detected in ST117 isolates were CT469 (*n* = 4), CT36 (*n* = 4), CT2614 (*n* = 1), CT2615 (*n* = 1) and CT1473 (*n* = 1).

**Fig. 2:**
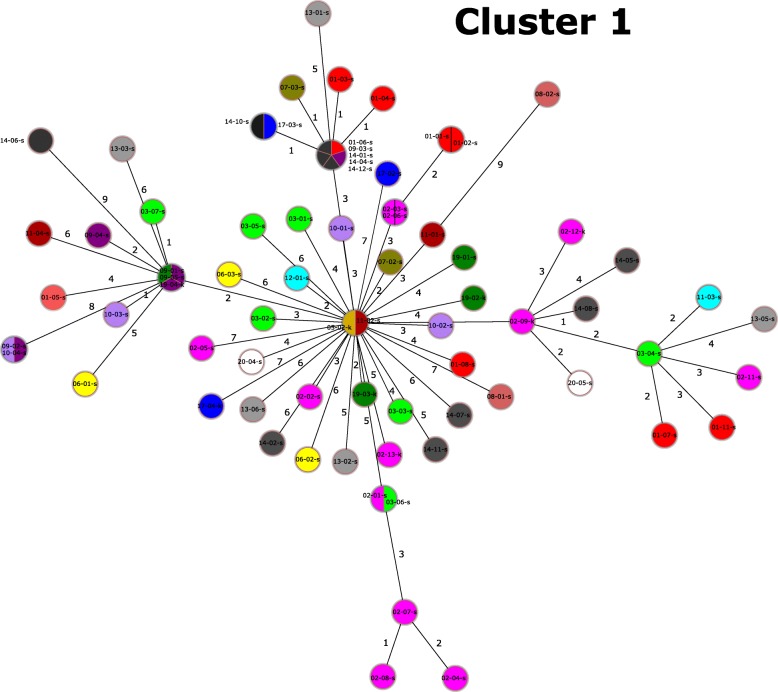
Minimum spanning tree of Cluster 1 based on cgMLST information. Different colours depict different clinics. Cluster distance threshold was set to 10 cgMLST alleles. Size of circles is representative and reflects the number of isolates

Of the CT71 isolates, 74/78 clustered into one cgMLST cluster type (Fig. 2, Cluster 1), that exhibited up to 10 cgMLST allele differences. All isolates of Cluster 1 harbored an identical insertion of a *van*B-encoding Tn*1549*-like transposon into a gene of unknown function (HMPREF0351_10592), previously reported for the *van*B-encoding VREfm of the sequence type ST192 [11]. Thus, these isolates constitute a clone, which we designate as the ST117/CT71/*van*B clone.

Statistical analyses using the Mann-Whitney U test [12] did not support an association of the CT71 clone with any of the patient data characteristics detailed in the Table 1 (Additional file 3: Table S3).

## Discussion

Surveillance, hygiene/infection control programs and antibiotic stewardship interventions for VREfm have been implemented in healthcare settings throughout Europe [13, 14]. However, the impact of these measures on the VRE influx by individual patients (colonization) entering the healthcare system is poorly understood. Here we characterized VREfm isolated from rectal swabs of patients during admission with and without prior history of hospitalization within the so-called “VRE-belt” in central Germany. Core genome-based phylogenetic analysis classifies all of the VREfm from this study as members of the hospital-associated clade A1 (data not shown) and shows that a single ST117 clonal lineage, with cgMLST complex type CT71 is predominant in the Rhine-Main metropolitan area within a patient population with a high-risk profile for VREfm acquisition. Previous epidemiological data indicated that the emergence of ST117 with its three major CTs, CT36, CT71 and CT469 is recent, and presently accounts for over one-third of all VREfm isolated from bloodstream infections in Germany and the Netherlands [15].

The repeated isolation of a single predominant ST117/CT71/*van*B clone at geographically separated institutions (Additional file 5: Figure S1) throughout the reporting period suggests that it has highly adaptive properties for effective transmission and a capacity for persistence in the hospital environment. Indeed, ST117/CT71/*van*B VREfm isolates harbored elements associated with persistence, such as the collagen-binding *acm* and the enterococcal surface protein Esp, required for promoting biofilm formation (Additional file 2: Table S2). In addition, the PTS_clin_ phosphotransferase system associated with colonization potential of clinical isolates [7] was present in all ST117/CT71/*van*B isolates. Further studies are warranted to understand the impact of these genes in the distribution of the ST117/CT71/*van*B clone.

A limitation of the study is the relatively short collection period and the number of isolates analyzed. Nevertheless, the discovery of a single predominant clone in geographically separated individual institutions within such a large catchment area is unprecedented. There are several possible explanations for this phenomenon: Firstly, our data indicate ongoing inter-hospital spread or even a multihospital outbreak, as near-identical isolates (≤10 cgMLST alleles) of the ST117/CT71/*van*B clone were detected in different hospitals (Fig. 2, Additional file 6: Figure S2). The latter phenomenon would require the movement of patients among the different hospitals sampled. This is true even for the smallest group of patients included in this study, i.e. those who have been reported to have previous stays in other participating hospitals (Additional file 6: Figure S2, Additional file 4: Table S4). Secondly, 14.9% (11/74) of the patients harboring this clone did not report any previous hospital stay within the last 12 months (Table 1). This indicates either acquisition of the VREfm in a hospital before more than 12 months ago, a nosocomial acquisition during the current hospital stay, or an acquisition through the dissemination of this clone in communal spaces outside of healthcare institutions. Further studies are required to answer the questions raised here, with particular focus on the presence of this clone in the community, healthcare-independent populations and other reservoirs (livestock, food, water).

## Conclusion

We report the detection of a near-ubiquitous VREfm clone (ST117/CT71/*van*B) circulating within the metropolitan region in and around Frankfurt am Main/Germany. The presence both of a single clone in such a large catchment area and the detection of a possible multi-hospital VRE transmission in this study has only been revealed as a result of WGS-based analysis. As vancomycin resistance is associated with enhanced mortality among patients in hospital settings, in particular bloodstream infections [16, 17], the prevention of VREfm infections is a major objective. The presence of a VREfm clone within different institutions questions whether infection control and antimicrobial stewardship interventions can be effective without an understanding of the VREfm carriage state and transmission dynamics in human populations within the catchment area studied. The results of our study call for the establishment of a multihospital infection control approach, including rapid detection tools to identify predominant clones and for a genome-based long-term surveillance to be able to detect newly emerging clones. In addition, the use of clone-based strategies for eradication i.e. based on vaccines or bacteriophages, would be interesting avenues for further pursuit.

## Additional files

Additional file 1: **Table S1.** Characteristics of the sequenced isolates. Depicts the characteristics of each VREfm isolate presented in this study. (DOCX 14 kb)

Additional file 2: **Table S2.** Characteristics of the participating hospitals. Depicts selected characteristics of the hospitals participating in the study. (DOCX 30 kb)

Additional file 3: **Table S3.** Statistical analysis of parameters associated with ST117/CT71/*van*B clone carriage. Depicts the statistical analysis of parameters associated with the carriage of the ST117/CT71/*van*B clone. (DOCX 12 kb)

Additional file 4: **Table S4.** Information on previous hospital stays of the patients. Depicts previous hospital stays of the patients, including information in which hospitals they previously resided. (DOCX 17 kb)

Additional file 5: **Figure S1.** Regional distribution of the Cluster 1 VREfm ST117/CT71/*van*B isolates. Depicts the regional distribution of Cluster 1 VREfm ST117/CT71/*van*B isolates. Districts may include more than one hospital. The original map was extracted from Googlemaps (https://www.google.de/maps/@50.2354853,8.7072805,11z). (PDF 2079 kb)

Additional file 6: **Figure S2.** Interaction map between the different participating hospitals. Indicates the patients’ previous hospital history, whereever known. Connections between hospitals mark previous hospital stays in another hospital, while circles indicate a previous stay in the same hospital. (PDF 15 kb)

## Data Availability

The raw sequencing data are available in ENA under the accession number PRJEB29744.

## Abbreviations

cgMLST: Core genome multilocus sequence typing
CT: Complex type
MDRO: Multidrug-resistant organisms
MLST: Multilocus sequence typing
ST: Sequence type
VRE: Vancomycin-resistant enterococci
VREfm: Vancomycin-resistant *Enterococcus faecium*
WGS: Whole-genome sequencing
WHO: World Health Organization
