# Supplementary material for: Near-ubiquitous presence of a vancomycin-resistant Enterococcus faecium ST117/CT71/vanB –clone in the Rhine-Main metropolitan area of Germany
Source: Antimicrob Resist Infect Control. 2019 Jul 29;8:128. doi: 10.1186/s13756-019-0573-8 (PMC6664515; doi:10.1186/s13756-019-0573-8)
Supplement: Supplementary file 1 — Table S1. Characteristics of the sequenced isolates. Depicts the characteristics of each VREfm isolate presented in this study. (DOCX 14 kb) [file 13756_2019_573_MOESM1_ESM.docx]

**Additional table 1: Characteristics of the participating hospitals.**

| **ID of hospital** | **Amount of VRE analysed** | **Beds/1 Isolate** | **type of hospital** |
| --- | --- | --- | --- |
| 1 | 13 | 114.5 | tertiary care |
| 2 | 12 | 48.5 | tertiary care |
| 3 | 7 | 144.6 | tertiary care |
| 4 | 3 | 130.0 | tertiary care |
| 5 | 3 | 118.7 | tertiary care |
| 6 | 3 | 232.3 | tertiary care |
| 7 | 3 | 81.7 | general practitioner's hospital |
| 8 | 3 | 108.7 | standard care |
| 9 | 5 | 205.4 | tertiary care |
| 10 | 4 | 82.8 | tertiary care |
| 11 | 4 | 118.5 | tertiary care |
| 12 | 2 | 50.0 | standard care |
| 13 | 8 | 48.1 | general practitioner's hospital |
| 14 | 11 | 88.0 | tertiary care |
| 17 | 4 | 66.8 | specialized clinic |
| 19 | 4 | 114.0 | standard care |
| 20 | 5 | 149.4 | tertiary care |
