# Supplementary material for: Near-ubiquitous presence of a vancomycin-resistant Enterococcus faecium ST117/CT71/vanB –clone in the Rhine-Main metropolitan area of Germany
Source: Antimicrob Resist Infect Control. 2019 Jul 29;8:128. doi: 10.1186/s13756-019-0573-8 (PMC6664515; doi:10.1186/s13756-019-0573-8)
Supplement: Supplementary file 2 — Table S2. Characteristics of the participating hospitals. Depicts selected characteristics of the hospitals participating in the study. (DOCX 30 kb) [file 13756_2019_573_MOESM2_ESM.docx]

**Additional Table 2: Characteristics of the sequenced isolates. Prediction of resistance and virulence genes was performed using goseqit (**[**https://www.goseqit.com/**](https://www.goseqit.com/)**) and manual blast of *esp* and PTS_clin_.**

| **Isolate ID** | **Hospital ID** | **Source^#^** | **ST** | **CT** | **Clade 1**  **CT71?** | **Integration**  **Site of *van**** | **Antibiotic resistance genes** | **Virulence genes** |
| --- | --- | --- | --- | --- | --- | --- | --- | --- |
| VRE-01-01-s | 1 | R | 117 | 71 | Yes | 10592 | *msr(C), erm(B), dfrG, vanB* | *hylEfm, acm, efaAfm, PTSclin, esp* |
| VRE-01-02-s | 1 | R | 117 | 71 | Yes | 10592 | *msr(C), erm(B), dfrG, vanB* | *hylEfm, acm, efaAfm, PTSclin, esp* |
| VRE-01-03-s | 1 | R | 117 | 71 | Yes | 10592 | *msr(C), erm(B), dfrG, vanB* | *hylEfm, acm, efaAfm, PTSclin, esp* |
| VRE-01-04-s | 1 | R | 117 | 71 | Yes | 10592 | *msr(C), erm(B), dfrG, vanB* | *hylEfm, acm, efaAfm, PTSclin, esp* |
| VRE-01-05-s | 1 | R | 117 | 71 | Yes | 10592 | *msr(C), erm(B), dfrG, vanB* | *hylEfm, acm, efaAfm, PTSclin, esp* |
| VRE-01-06-s | 1 | R | 117 | 71 | Yes | 10592 | *msr(C), erm(B), dfrG, vanB* | *hylEfm, acm, efaAfm, PTSclin, esp* |
| VRE-01-07-s | 1 | R | 117 | 71 | Yes | 10592 | *msr(C), erm(B), dfrG, vanB* | *hylEfm, acm, efaAfm, PTSclin, esp* |
| VRE-01-08-s | 1 | R | 117 | 71 | Yes | 10592 | *msr(C), erm(B), dfrG, vanB* | *hylEfm, acm, efaAfm, PTSclin, esp* |
| VRE-01-09-s | 1 | R | 117 | 71 | No | 10592 | *msr(C), erm(B), dfrG, vanB* | *hylEfm, acm, efaAfm, PTSclin, esp* |
| VRE-01-10-s | 1 | R | 117 | 36 | N.A.^$^ | araA | *ant(6)-Ia, aph(3')-III, erm(B), msr(C), dfrG, vanB* | *hylEfm, acm, efaAfm, PTSclin, esp* |
| VRE-01-11-s | 1 | R | 117 | 71 | Yes | 10592 | *msr(C), erm(B), dfrG, vanB* | *hylEfm, acm, efaAfm, PTSclin, esp* |
| VRE-01-12-s | 1 | R | 117 | 71 | No | 10592 | *msr(C), erm(B), dfrG, vanB* | *hylEfm, acm, efaAfm, PTSclin, esp* |
| VRE-01-13-s | 1 | R | 262 | 2613 | N.A.^$^ | N.D.^§^ | *msr(C), vanA* | *hylEfm, acm, efaAfm* |
| VRE-02-01-s | 2 | R | 117 | 71 | Yes | 10592 | *msr(C), erm(B), dfrG, vanB* | *hylEfm, acm, efaAfm, PTSclin, esp* |
| VRE-02-02-s | 2 | R | 117 | 71 | Yes | 10592 | *msr(C), erm(B), dfrG, vanB* | *hylEfm, acm, efaAfm, PTSclin, esp* |
| VRE-02-03-s | 2 | R | 117 | 71 | Yes | 10592 | *msr(C), erm(B), dfrG, vanB* | *hylEfm, acm, efaAfm, PTSclin, esp* |
| VRE-02-04-s | 2 | R | 117 | 71 | Yes | 10592 | *msr(C), erm(B), dfrG, vanB* | *hylEfm, acm, efaAfm, PTSclin, esp* |
| VRE-02-05-s | 2 | R | 117 | 71 | Yes | 10592 | *msr(C), erm(B), dfrG, vanB* | *hylEfm, acm, efaAfm, PTSclin, esp* |
| VRE-02-06-s | 2 | R | 117 | 71 | Yes | 10592 | *msr(C), erm(B), dfrG, vanB* | *hylEfm, acm, efaAfm, PTSclin, esp* |
| VRE-02-07-s | 2 | R | 117 | 71 | Yes | 10592 | *msr(C), erm(B), dfrG, vanB* | *hylEfm, acm, efaAfm, PTSclin, esp* |
| VRE-02-08-s | 2 | R | 117 | 71 | Yes | 10592 | *msr(C), erm(B), dfrG, vanB* | *hylEfm, acm, efaAfm, PTSclin, esp* |
| VRE-02-09-k | 2 | C | 117 | 71 | Yes | 10592 | *msr(C), erm(B), dfrG, vanB* | *hylEfm, acm, efaAfm, PTSclin, esp* |
| VRE-02-11-s | 2 | R | 117 | 71 | Yes | 10592 | *msr(C), erm(B), dfrG, vanB* | *hylEfm, acm, efaAfm, PTSclin, esp* |
| VRE-02-12-k | 2 | C | 117 | 71 | Yes | 10592 | *msr(C), erm(B), dfrG, vanB* | *hylEfm, acm, efaAfm, PTSclin, esp* |
| VRE-02-13-k | 2 | C | 117 | 71 | Yes | 10592 | *msr(C), erm(B), dfrG, vanB* | *hylEfm, acm, efaAfm, PTSclin, esp* |
| VRE-03-01-s | 3 | R | 117 | 71 | Yes | 10592 | *msr(C), erm(B), dfrG, vanB* | *hylEfm, acm, efaAfm, PTSclin, esp* |
| VRE-03-02-s | 3 | R | 117 | 71 | Yes | 10592 | *msr(C), erm(B), dfrG, vanB* | *hylEfm, acm, efaAfm, PTSclin, esp* |
| VRE-03-03-s | 3 | R | 117 | 71 | Yes | 10592 | *msr(C), erm(B), dfrG, vanB* | *hylEfm, acm, efaAfm, PTSclin, esp* |
| VRE-03-04-s | 3 | R | 117 | 71 | Yes | 10592 | *msr(C), erm(B), dfrG, vanB* | *hylEfm, acm, efaAfm, PTSclin, esp* |
| VRE-03-05-s | 3 | R | 117 | 71 | Yes | 10592 | *msr(C), erm(B), dfrG, vanB* | *hylEfm, acm, efaAfm, PTSclin, esp* |
| VRE-03-06-s | 3 | R | 117 | 71 | Yes | 10592 | *msr(C), erm(B), dfrG, vanB* | *hylEfm, acm, efaAfm, PTSclin, esp* |
| VRE-03-07-s | 3 | R | 117 | 71 | Yes | 10592 | *msr(C), erm(B), dfrG, vanB* | *hylEfm, acm, efaAfm, PTSclin, esp* |
| VRE-04-01-s | 4 | R | 117 | 36 | N.A.^$^ | 10618 | *aph(3')-III, ant(6)-Ia, erm(B), msr(C), dfrG, vanB* | *hylEfm, acm, efaAfm* |
| VRE-04-02-s | 4 | R | 80 | 899 | N.A.^$^ | N.D.^§^ | *erm(B), erm(T), msr(C), dfrG, tet(L), tet(M), vanB* | *hylEfm, acm, efaAfm, PTSclin* |
| VRE-04-03-s | 4 | R | 117 | 36 | N.A.^$^ | araA | *ant(6)-Ia, aph(3')-III, erm(B), msr(C), dfrG, vanB* | *hylEfm, acm, efaAfm, PTSclin* |
| VRE-05-01-s | 5 | R | 117 | 469 | N.A.^$^ | 10592 | *ant(6)-Ia, aph(3')-III, erm(B), msr(C), dfrG, vanB* | *hylEfm, acm, efaAfm, PTSclin, esp* |
| VRE-05-02-k | 5 | C | 117 | 71 | Yes | 10592 | *aac(6')-aph(2''), erm(B), msr(C), dfrG, vanB* | *hylEfm, acm, efaAfm, PTSclin, esp* |
| VRE-05-03-s | 5 | R | 117 | 469 | N.A.^$^ | 10592 | *ant(6)-Ia, aph(3')-III, erm(B), msr(C), dfrG, vanB* | *hylEfm, acm, efaAfm, PTSclin, esp* |
| VRE-06-01-s | 6 | R | 117 | 71 | Yes | 10592 | *msr(C), erm(B), dfrG, vanB* | *hylEfm, acm, efaAfm, PTSclin, esp* |
| VRE-06-02-s | 6 | R | 117 | 71 | Yes | 10592 | *msr(C), erm(B), dfrG, vanB* | *hylEfm, acm, efaAfm, PTSclin, esp* |
| VRE-06-03-s | 6 | R | 117 | 71 | Yes | 10592 | *msr(C), erm(B), dfrG, vanB* | *hylEfm, acm, efaAfm, PTSclin, esp* |
| VRE-07-01-s | 7 | R | 117 | 469 | N.A.^$^ | 10592 | *ant(6)-Ia, aph(3')-III, erm(B), msr(C), dfrG, vanB* | *hylEfm, acm, efaAfm, PTSclin, esp* |
| VRE-07-02-s | 7 | R | 117 | 71 | Yes | 10592 | *msr(C), erm(B), dfrG, vanB* | *hylEfm, acm, efaAfm, PTSclin, esp* |
| VRE-07-03-s | 7 | R | 117 | 71 | Yes | 10592 | *msr(C), erm(B), dfrG, vanB* | *hylEfm, acm, efaAfm, PTSclin, esp* |
| VRE-08-01-s | 8 | R | 117 | 71 | Yes | 10592 | *msr(C), erm(B), dfrG, vanB* | *hylEfm, acm, efaAfm, PTSclin, esp* |
| VRE-08-02-s | 8 | R | 117 | 71 | Yes | 10592 | *msr(C), erm(B), dfrG, vanB* | *hylEfm, acm, efaAfm, PTSclin, esp* |
| VRE-08-03-s | 8 | R | 192 | 10 | N.A.^$^ | 10592 | *ant(6)-Ia, aph(3')-III, msr(C), erm(B), lnu(B),*  *tet(M), tet(L), vanB* | *acm, efaAfm, PTSclin, esp* |
| VRE-09-01-s | 9 | R | 117 | 71 | Yes | 10592 | *msr(C), erm(B), dfrG, vanB* | *hylEfm, acm, efaAfm, PTSclin, esp* |
| VRE-09-02-s | 9 | R | 117 | 71 | Yes | 10592 | *msr(C), erm(B), dfrG, vanB* | *hylEfm, acm, efaAfm, PTSclin, esp* |
| VRE-09-03-s | 9 | R | 117 | 71 | Yes | 10592 | *msr(C), erm(B), dfrG, vanB* | *hylEfm, acm, efaAfm, PTSclin, esp* |
| VRE-09-04-s | 9 | R | 117 | 71 | Yes | 10592 | *msr(C), erm(B), dfrG, vanB* | *hylEfm, acm, efaAfm, PTSclin, esp* |
| VRE-09-05-s | 9 | R | 117 | 71 | Yes | 10592 | *msr(C), erm(B), dfrG, vanB* | *hylEfm, acm, efaAfm, PTSclin, esp* |
| VRE-10-01-s | 10 | R | 117 | 71 | Yes | 10592 | *msr(C), erm(B), dfrG, vanB* | *hylEfm, acm, efaAfm, PTSclin, esp* |
| VRE-10-02-s | 10 | R | 117 | 71 | Yes | 10592 | *msr(C), erm(B), dfrG, vanB* | *hylEfm, acm, efaAfm, PTSclin, esp* |
| VRE-10-03-s | 10 | R | 117 | 71 | Yes | 10592 | *msr(C), erm(B), dfrG, vanB* | *hylEfm, acm, efaAfm, PTSclin, esp* |
| VRE-10-04-s | 10 | R | 117 | 71 | Yes | 10592 | *msr(C), erm(B), dfrG, vanB* | *hylEfm, acm, efaAfm, PTSclin, esp* |
| VRE-11-01-s | 11 | R | 117 | 71 | Yes | 10592 | *msr(C), erm(B), dfrG, vanB* | *hylEfm, acm, efaAfm, PTSclin, esp* |
| VRE-11-02-s | 11 | R | 117 | 71 | Yes | 10592 | *msr(C), erm(B), dfrG, vanB* | *hylEfm, acm, efaAfm, PTSclin, esp* |
| VRE-11-03-s | 11 | R | 117 | 71 | Yes | 10592 | *msr(C), erm(B), dfrG, vanB* | *hylEfm, acm, efaAfm, PTSclin, esp* |
| VRE-11-04-s | 11 | R | 117 | 71 | Yes | 10592 | *msr(C), erm(B), dfrG, vanB* | *hylEfm, acm, efaAfm, PTSclin, esp* |
| VRE-12-01-s | 12 | R | 117 | 71 | Yes | 10592 | *msr(C), erm(B), dfrG, vanB* | *hylEfm, acm, efaAfm, PTSclin, esp* |
| VRE-12-03-s | 12 | R | 117 | 2614 | N.A.^$^ | 10592 | *msr(C), erm(B), dfrG, vanB* | *hylEfm, acm, efaAfm, PTSclin, esp* |
| VRE-13-01-s | 13 | R | 117 | 71 | Yes | 10592 | *msr(C), erm(B), dfrG, vanB* | *hylEfm, acm, efaAfm, PTSclin, esp* |
| VRE-13-02-s | 13 | R | 117 | 71 | Yes | 10592 | *msr(C), erm(B), dfrG, vanB* | *hylEfm, acm, efaAfm, PTSclin, esp* |
| VRE-13-03-s | 13 | R | 117 | 71 | Yes | 10592 | *aac(6')-aph(2''), erm(B), msr(C), dfrG, vanB* | *hylEfm, acm, efaAfm, PTSclin, esp* |
| VRE-13-04-s | 13 | R | 117 | 71 | No | 10592 | *msr(C), erm(B), dfrG, vanB* | *hylEfm, acm, efaAfm, PTSclin, esp* |
| VRE-13-05-s | 13 | R | 117 | 71 | Yes | 10592 | *msr(C), erm(B), dfrG, vanB* | *hylEfm, acm, efaAfm, PTSclin, esp* |
| VRE-13-06-s | 13 | R | 117 | 71 | Yes | 10592 | *msr(C), erm(B), dfrG, vanB* | *hylEfm, acm, efaAfm, PTSclin, esp* |
| VRE-13-07-s | 13 | R | 117 | 71 | No | 10592 | *msr(C), erm(B), dfrG, vanB* | *hylEfm, acm, efaAfm, PTSclin, esp* |
| VRE-13-08-s | 13 | R | 117 | Q# | N.A.^$^ | 10592 | *msr(C), erm(B), dfrG, vanB* | *hylEfm, acm, efaAfm, PTSclin, esp* |
| VRE-14-01-s | 14 | R | 117 | 71 | Yes | 10592 | *msr(C), erm(B), dfrG, vanB* | *hylEfm, acm, efaAfm, PTSclin, esp* |
| VRE-14-02-s | 14 | R | 117 | 71 | Yes | 10592 | *msr(C), erm(B), dfrG, vanB* | *hylEfm, acm, efaAfm, PTSclin, esp* |
| VRE-14-03-k | 14 | C | 117 | 2615 | N.A.^$^ | 10592 | *msr(C), erm(B), dfrG, vanB* | *hylEfm, acm, efaAfm, PTSclin, esp* |
| VRE-14-04-s | 14 | R | 117 | 71 | Yes | 10592 | *msr(C), erm(B), dfrG, vanB* | *hylEfm, acm, efaAfm, PTSclin, esp* |
| VRE-14-05-s | 14 | R | 117 | 71 | Yes | 10592 | *msr(C), erm(B), dfrG, vanB* | *hylEfm, acm, efaAfm, PTSclin, esp* |
| VRE-14-06-s | 14 | R | 117 | 71 | Yes | 10592 | *msr(C), erm(B), dfrG, vanB* | *hylEfm, acm, efaAfm, PTSclin, esp* |
| VRE-14-07-s | 14 | R | 117 | 71 | Yes | 10592 | *msr(C), erm(B), dfrG, vanB* | *hylEfm, acm, efaAfm, PTSclin, esp* |
| VRE-14-08-s | 14 | R | 117 | 71 | Yes | 10592 | *msr(C), erm(B), dfrG, vanB* | *hylEfm, acm, efaAfm, PTSclin, esp* |
| VRE-14-10-s | 14 | R | 117 | 71 | Yes | 10592 | *msr(C), erm(B), dfrG, vanB* | *hylEfm, acm, efaAfm, PTSclin, esp* |
| VRE-14-11-s | 14 | R | 117 | 71 | Yes | 10592 | *msr(C), erm(B), dfrG, vanB* | *hylEfm, acm, efaAfm, PTSclin, esp* |
| VRE-14-12-s | 14 | R | 117 | 71 | Yes | 10592 | *msr(C), erm(B), dfrG, vanB* | *hylEfm, acm, efaAfm, PTSclin, esp* |
| VRE-17-02-s | 17 | R | 117 | 71 | Yes | 10592 | *msr(C), dfrG, vanB* | *hylEfm, acm, efaAfm, PTSclin, esp* |
| VRE-17-03-s | 17 | R | 117 | 71 | Yes | 10592 | *msr(C), erm(B), dfrG, vanB* | *hylEfm, acm, efaAfm, PTSclin, esp* |
| VRE-17-04-k | 17 | C | 117 | 71 | Yes | 10592 | *msr(C), erm(B), dfrG, vanB* | *hylEfm, acm, efaAfm, PTSclin, esp* |
| VRE-17-05-s | 17 | R | 117 | 469 | N.A.^$^ | 10592 | *ant(6)-Ia, aph(3')-III, erm(B), msr(C), dfrG, vanB* | *hylEfm, acm, efaAfm, PTSclin, esp* |
| VRE-19-01-s | 19 | R | 117 | 71 | Yes | 10592 | *msr(C), erm(B), dfrG, vanB* | *hylEfm, acm, efaAfm, PTSclin, esp* |
| VRE-19-02-k | 19 | C | 117 | 71 | Yes | 10592 | *msr(C), erm(B), dfrG, vanB* | *hylEfm, acm, efaAfm, PTSclin, esp* |
| VRE-19-03-k | 19 | C | 117 | 71 | Yes | 10592 | *msr(C), erm(B), dfrG, vanB* | *hylEfm, acm, efaAfm, PTSclin, esp* |
| VRE-19-04-k | 19 | C | 117 | 71 | Yes | 10592 | *msr(C), erm(B), dfrG, vanB* | *hylEfm, acm, efaAfm, PTSclin, esp* |
| VRE-20-01-s | 20 | R | 117 | 36 | N.A.^$^ | araA | *ant(6)-Ia, aph(3')-III, aac(6')-aph(2''), erm(B),*  *msr(C), dfrG, vanB* | *hylEfm, acm, efaAfm, PTSclin, esp* |
| VRE-20-02-s | 20 | R | 117 | 1473 | N.A.^$^ | 10592 | *msr(C), erm(B), dfrG, vanB* | *hylEfm, acm, efaAfm, PTSclin, esp* |
| VRE-20-03-s | 20 | R | 1428 | 2616 | N.A.^$^ | 10592 | *msr(C), dfrG, vanB* | *hylEfm, acm, efaAfm, PTSclin, esp* |
| VRE-20-04-s | 20 | R | 117 | 71 | Yes | 10592 | *msr(C), erm(B), dfrG, vanB* | *acm, efaAfm, PTSclin, esp* |
| VRE-20-05-s | 20 | R | 117 | 71 | Yes | 10592 | *msr(C), erm(B), dfrG, vanB* | *hylEfm, acm, efaAfm, PTSclin, esp* |

Source, R= rectal swab, C= Clinical isolate; N.A.^$^, not applicable; *position according to *Enterococcus faecium* DO (accession number NC_017960); N.D.^§^, not determined. Q# , this isolate harboured only 70% of the cgMLST alleles (even though the sequencing was ok) and was therefore excluded from the cgMLST analysis.
