# Supplementary material for: Near-ubiquitous presence of a vancomycin-resistant Enterococcus faecium ST117/CT71/vanB –clone in the Rhine-Main metropolitan area of Germany
Source: Antimicrob Resist Infect Control. 2019 Jul 29;8:128. doi: 10.1186/s13756-019-0573-8 (PMC6664515; doi:10.1186/s13756-019-0573-8)
Supplement: Supplementary file 3 — Table S3. Statistical analysis of parameters associated with ST117/CT71/vanB clone carriage. Depicts the statistical analysis of parameters associated with the carriage of the ST117/CT71/vanB clone. (DOCX 12 kb) [file 13756_2019_573_MOESM3_ESM.docx]

| **Parameter** | **Mann-Whitney-U** | **Wilcoxon-W** | **Z** | **P-value** |
| --- | --- | --- | --- | --- |
| Sex | 384 | 475 | -1.629 | 0.103 |
| Age | 448 | 3529 | -0.670 | 0.503 |
| Underlying disease | 234 | 289 | -1.200 | 0.230 |
| Traveling aboard | 385 | 476 | -1.272 | 0.203 |
| Previous hospital stay | 470 | 561 | -0.295 | 0.768 |
| Previous antimicrobial therapy | 461 | 3387 | -0.511 | 0.610 |

**Additional table 3: Statistical analysis of parameters associated with ST117/CT71 carriage.**
