# Supplementary material for: Near-ubiquitous presence of a vancomycin-resistant Enterococcus faecium ST117/CT71/vanB –clone in the Rhine-Main metropolitan area of Germany
Source: Antimicrob Resist Infect Control. 2019 Jul 29;8:128. doi: 10.1186/s13756-019-0573-8 (PMC6664515; doi:10.1186/s13756-019-0573-8)
Supplement: Supplementary file 4 — Table S4. Information on previous hospital stays of the patients. Depicts previous hospital stays of the patients, including information in which hospitals they previously resided. (DOCX 17 kb) [file 13756_2019_573_MOESM4_ESM.docx]

**Additional Table 4: Information on previous hospital stays of the patients. Other refers to a previous stay in a hospital that was not participating in the study. Source, R= rectal swab, C= Clinical isolate**

| **Isolate ID** | **Source** | **Hospital ID** | **VRE previously detected** | **Previous hospital stay** | **Previous hospital stay in hospital No.** |
| --- | --- | --- | --- | --- | --- |
| VRE-01-01-s | R | 1 | No | Yes | 2 |
| VRE-01-02-s | R | 1 | No | Not ascertainable | Not known |
| VRE-01-03-s | R | 1 | No | None | None |
| VRE-01-04-s | R | 1 | No | Yes | Other |
| VRE-01-05-s | R | 1 | No | Yes | 1 |
| VRE-01-06-s | R | 1 | No | Yes | Other |
| VRE-01-07-s | R | 1 | No | Yes | 14 |
| VRE-01-08-s | R | 1 | No | Yes | Other |
| VRE-01-09-s | R | 1 | Yes | Yes | 1 |
| VRE-01-10-s | R | 1 | No | Yes | 5 |
| VRE-01-11-s | R | 1 | No | Yes | 1 |
| VRE-01-12-s | R | 1 | No | Yes | Other |
| VRE-01-13-s | R | 1 | Yes | Yes | 1 |
| VRE-02-01-s | R | 2 | Yes | Yes | 2 |
| VRE-02-02-s | R | 2 | No | Yes | Other |
| VRE-02-03-s | R | 2 | No | Yes | 2 |
| VRE-02-04-s | R | 2 | No | Yes | 2 |
| VRE-02-05-s | R | 2 | No | Yes | 2 |
| VRE-02-06-s | R | 2 | No | Yes | 2 |
| VRE-02-07-s | R | 2 | No | None | None |
| VRE-02-08-s | R | 2 | No | None | None |
| VRE-02-09-k | C | 2 | No | Yes | 6 |
| VRE-02-11-s | R | 2 | No | Yes | 3 |
| VRE-02-12-k | C | 2 | No | Yes | 2 |
| VRE-02-13-k | C | 2 | Yes | Yes | 3 |
| VRE-03-01-s | R | 3 | No | Yes | 3 |
| VRE-03-02-s | R | 3 | No | None | None |
| VRE-03-03-s | R | 3 | No | Yes | 1, 11 |
| VRE-03-04-s | R | 3 | No | Yes | 1, 8 |
| VRE-03-05-s | R | 3 | No | Yes | 6, other |
| VRE-03-06-s | R | 3 | No | Yes | 2 |
| VRE-03-07-s | R | 3 | Yes | Yes | 7, other |
| VRE-04-01-s | R | 4 | No | Yes | 4 |
| VRE-04-02-s | R | 4 | No | Yes | 4, 6, 7 |
| VRE-04-03-s | R | 4 | No | Yes | 4 |
| VRE-05-01-s | R | 5 | No | Yes | Other |
| VRE-05-02-k | C | 5 | No | Yes | Other |
| VRE-05-03-s | R | 5 | No | Yes | 5 |
| VRE-06-01-s | R | 6 | No | Yes | 6 |
| VRE-06-02-s | R | 6 | No | Yes | 6, 7 |
| VRE-06-03-s | R | 6 | No | Yes | 6, 7 |
| VRE-07-01-s | R | 7 | No | Yes | 8 |
| VRE-07-02-s | R | 7 | No | Yes | Other |
| VRE-07-03-s | R | 7 | No | Yes | 7, other |
| VRE-08-01-s | R | 8 | Yes | None | None |
| VRE-08-02-s | R | 8 | Yes | Yes | 8 |
| VRE-08-03-s | R | 8 | No | Yes | 8 |
| VRE-09-01-s | R | 9 | No | Yes | Other |
| VRE-09-02-s | R | 9 | No | Yes | 15 |
| VRE-09-03-s | R | 9 | Yes | Yes | Other |
| VRE-09-04-s | R | 9 | No | Yes | 9, other |
| VRE-09-05-s | R | 9 | No | Yes | Other |
| VRE-10-01-s | R | 10 | No | Yes | 13 |
| VRE-10-02-s | R | 10 | No | None | None |
| VRE-10-03-s | R | 10 | No | Yes | 10 |
| VRE-10-04-s | R | 10 | No | None | None |
| VRE-11-01-s | R | 11 | Yes | Yes | Other |
| VRE-11-02-s | R | 11 | Yes | Yes | 14, other |
| VRE-11-03-s | R | 11 | Yes | Yes | 8; other |
| VRE-11-04-s | R | 11 | Yes | Yes | 1, 6, 11 |
| VRE-12-01-s | R | 12 | Yes | Yes | 17, other |
| VRE-12-03-s | R | 12 | Yes | Yes | 11, other |
| VRE-13-01-s | R | 13 | No | Not ascertainable | Not known |
| VRE-13-02-s | R | 13 | No | Yes | Other |
| VRE-13-03-s | R | 13 | No | Not ascertainable | Not known |
| VRE-13-04-s | R | 13 | No | Yes | 13 |
| VRE-13-05-s | R | 13 | No | Yes | 3, other |
| VRE-13-06-s | R | 13 | No | Not ascertainable | Not known |
| VRE-13-07-s | R | 13 | No | Not ascertainable | Not known |
| VRE-13-08-s | R | 13 | No | Not ascertainable | Not known |
| VRE-14-01-s | R | 14 | No | Yes | 14 |
| VRE-14-02-s | R | 14 | No | Yes | Other |
| VRE-14-03-k | C | 14 | No | Yes | Other |
| VRE-14-04-s | R | 14 | No | None | None |
| VRE-14-05-s | R | 14 | No | Yes | 14 |
| VRE-14-06-s | R | 14 | No | Yes | Other |
| VRE-14-07-s | R | 14 | No | Yes | 14 |
| VRE-14-08-s | R | 14 | No | Yes | 14 |
| VRE-14-10-s | R | 14 | No | Yes | 11 |
| VRE-14-11-s | R | 14 | No | Yes | 14 |
| VRE-14-12-s | R | 14 | No | None | None |
| VRE-17-02-s | R | 17 | No | Yes | Other |
| VRE-17-03-s | R | 17 | No | Yes | Other |
| VRE-17-04-k | C | 17 | No | Yes | Other |
| VRE-17-05-s | R | 17 | No | Yes | Other |
| VRE-19-01-s | R | 19 | No | Yes | Other |
| VRE-19-02-k | C | 19 | No | None | None |
| VRE-19-03-k | C | 19 | No | None | None |
| VRE-19-04-k | C | 19 | No | Yes | 19 |
| VRE-20-01-s | R | 20 | No | Yes | 20 |
| VRE-20-02-s | R | 20 | No | Yes | 20 |
| VRE-20-03-s | R | 20 | No | None | None |
| VRE-20-04-s | R | 20 | No | Yes | 20 |
| VRE-20-05-s | R | 20 | No | Yes | 20 |
