# Supplementary figures and images for: Near-ubiquitous presence of a vancomycin-resistant Enterococcus faecium ST117/CT71/vanB –clone in the Rhine-Main metropolitan area of Germany
Source: Antimicrob Resist Infect Control. 2019 Jul 29;8:128. doi: 10.1186/s13756-019-0573-8 (PMC6664515; doi:10.1186/s13756-019-0573-8)

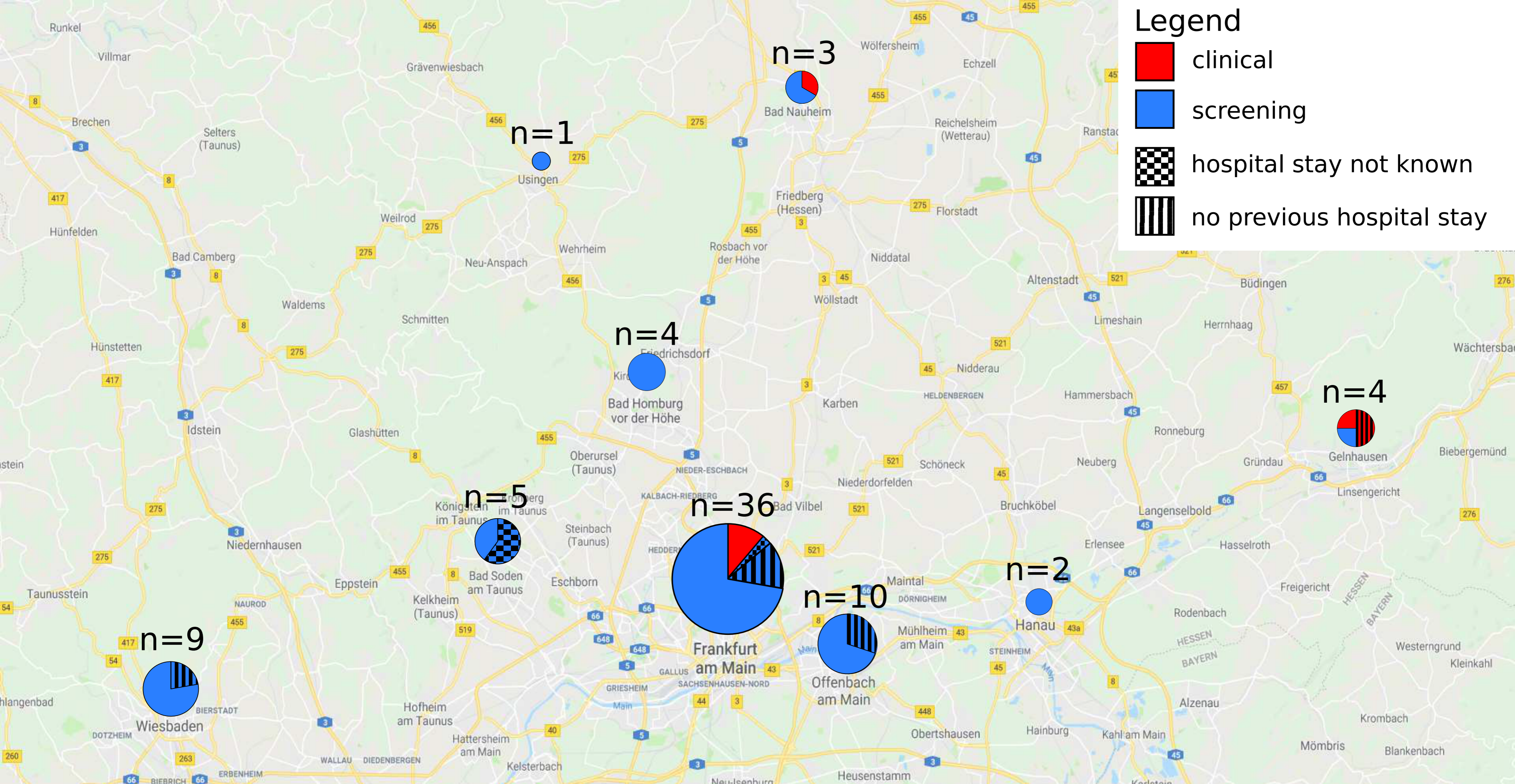

Supplement: Supplementary file 5 — Figure S1. Regional distribution of the Cluster 1 VREfm ST117/CT71/vanB isolates. Depicts the regional distribution of Cluster 1 VREfm ST117/CT71/vanB isolates. Districts may include more than one hospital. The original map was extracted from Googlemaps (https://www.google.de/maps/@50.2354853,8.7072805,11z). (PDF 2079 kb) [file 13756_2019_573_MOESM5_ESM.pdf]

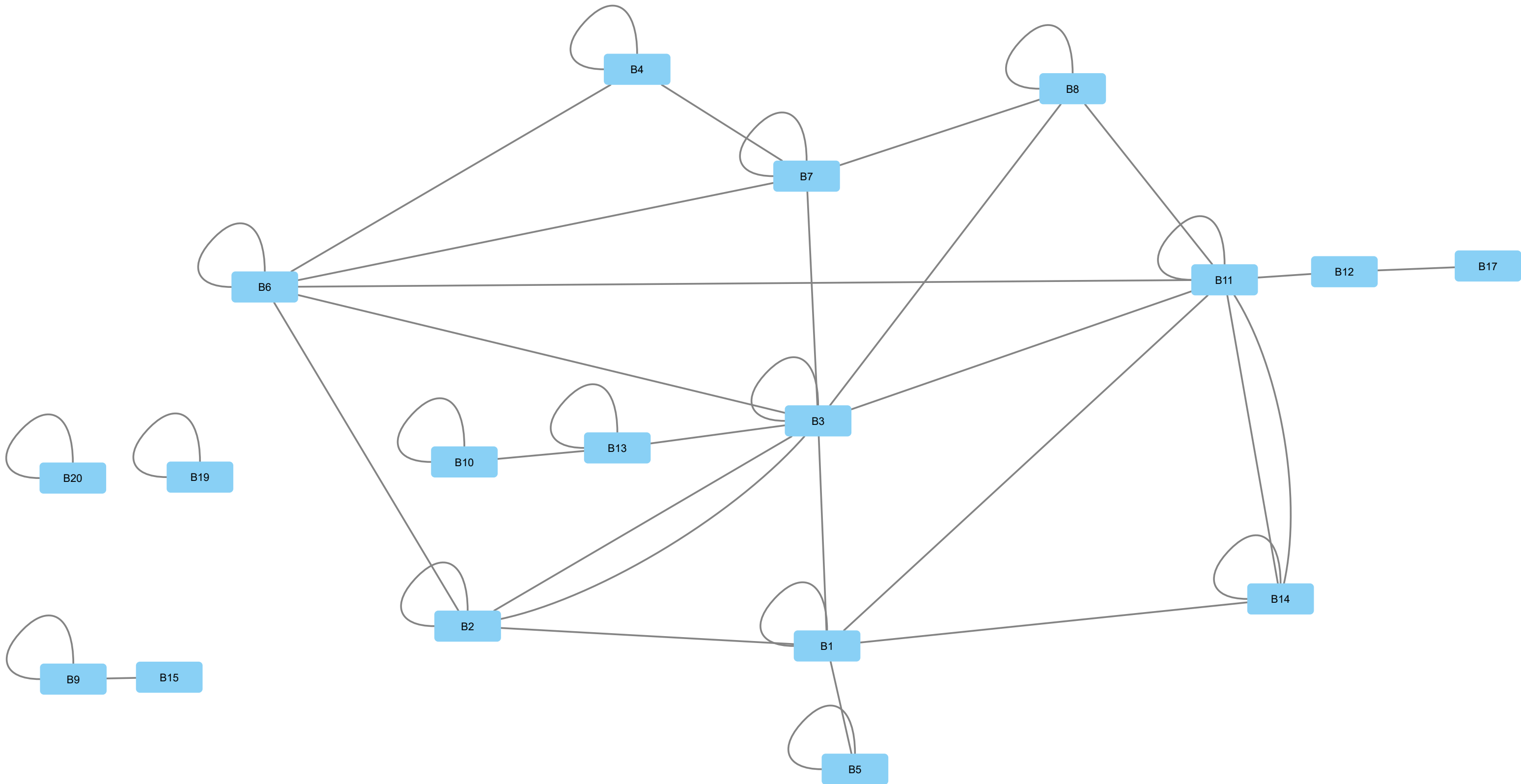

Supplement: Supplementary file 6 — Figure S2. Interaction map between the different participating hospitals. Indicates the patients’ previous hospital history, whereever known. Connections between hospitals mark previous hospital stays in another hospital, while circles indicate a previous stay in the same hospital. (PDF 15 kb) [file 13756_2019_573_MOESM6_ESM.pdf]
